# Therapeutic Response to Dihydroartemisinin–Piperaquine for *P. falciparum* and *P. vivax* Nine Years after Its Introduction in Southern Papua, Indonesia

**DOI:** 10.4269/ajtmh.17-0662

**Published:** 2018-01-15

**Authors:** Jeanne Rini Poespoprodjo, Enny Kenangalem, Johny Wafom, Freis Chandrawati, Agatha M. Puspitasari, Benedikt Ley, Leily Trianty, Zoé Korten, Asik Surya, Din Syafruddin, Nicholas M. Anstey, Jutta Marfurt, Rintis Noviyanti, Ric N. Price

**Affiliations:** 1 Mimika District Hospital, Timika, Indonesia;; 2 Timika Malaria Research Programme, Papuan Health and Community Development Foundation, Timika, Indonesia;; 3 Paediatric Research Office, Department of Child Health, Faculty of Medicine, Universitas Gadjah Mada/Dr. Sardjito Hospital, Yogyakarta, Indonesia;; 4 Eijkman Institute for Molecular Biology, Jakarta, Indonesia;; 5 Global and Tropical Health Division, Menzies School of Health Research and Charles Darwin University, Darwin, Australia;; 6 Indonesian Ministry of Health, National Malaria Control Program, Jakarta, Indonesia;; 7 Centre for Tropical Medicine and Global Health, Nuffield Department of Clinical Medicine, University of Oxford, Oxford, United Kingdom

## Abstract

Dihydroartemisinin–piperaquine (DHP) has been the first-line treatment of uncomplicated malaria due to both *Plasmodium falciparum* and *Plasmodium vivax* infections in Papua, Indonesia, since March 2006. The efficacy of DHP was reassessed to determine whether there had been any decline following almost a decade of its extensive use. An open-label drug efficacy study of DHP for uncomplicated *P. falciparum* and *P. vivax* malaria was carried out between March 2015 and April 2016 in Timika, Papua, Indonesia. Patients with uncomplicated malaria were administered supervised DHP tablets once daily for 3 days. Clinical and laboratory data were collected daily until parasite clearance and then weekly for 6 weeks. Molecular analysis was undertaken for all patients with recurrent parasitemia. A total of 129 study patients were enrolled in the study. At day 42, the polymerase chain reaction-adjusted efficacy was 97.7% (95% confidence intervals [CI]: 87.4–99.9) in the 61 patients with *P. falciparum* malaria, and 98.2% [95% CI: 90.3–100] in the 56 patients with *P. vivax* malaria. By day 2, 98% (56/57) of patients with *P. falciparum* and 96.9% (63/65) of those with *P. vivax* had cleared their peripheral parasitemia; none of the patients were still parasitaemic on day 3. Molecular analysis of *P. falciparum* parasites showed that none (0/61) had K13 mutations associated previously with artemisinin resistance or increased copy number of *plasmepsin* 2–3 (0/61). In the absence of artemisinin resistance, DHP has retained high efficacy for the treatment of uncomplicated malaria despite extensive drug pressure over a 9-year period.

## INTRODUCTION

Over the last 50 years, *Plasmodium falciparum* has developed resistance to chloroquine (CQ), mefloquine, sulphadoxine–pyrimethamine, piperaquine, and artemisinin, with the earliest evidence of resistance for all of these drugs documented in the Greater Mekong Subregion (GMS). By contrast, the epicenter of antimalarial drug resistance in *Plasmodium vivax* is on the island of Papua. In southern Papua, Indonesia, both *P. falciparum* and *P. vivax* malaria are equally prevalent.^1,2^ Clinical trials conducted in 2004 highlighted a very high risk of treatment failure following treatment with locally recommend antimalarial regimens. Within 28 days, 48% of patients with *P. falciparum* had failed treatment with CQ plus sulfadoxine–pyrimethamine (CQ + SP) and 65% of those with *P. vivax* had failed treatment with CQ monotherapy.^3^ In view of these findings, the first-line treatment of uncomplicated malaria due to either *P. falciparum* or *P. vivax* was changed to dihydroartemisinin–piperaquine (DHP) in March 2006. At the time of introduction of DHP, the risk of recurrent parasitemia within 42 days was 4.1% following *P. falciparum* and 10% following *P. vivax* malaria.^4^

Over the last 10 years, there has been growing concern regarding the emergence of artemisinin-resistant parasites in the GMS.^5–7^ The spread of these parasites to other locations has potential to undermine the global policy of artemisinin-based combination therapy (ACT) for uncomplicated malaria. More recently, there have been reports of declining efficacy of the partner drug piperaquine in areas where the prevalence of artemisinin resistance is high.^8^ The spread of these multidrug-resistant parasites eastward across the Indonesian archipelago poses a significant threat to the national antimalarial policy. In Papua, where DHP has been used extensively over the last 9 years, a clinical study was undertaken to assess whether it still retained excellent efficacy.

## MATERIALS AND METHODS

### Study site.

The study was carried out in Timika, southern Papua, Indonesia, between March 2015 and April 2016. The epidemiology of this area has been described previously.^1,9,10^ In brief, malaria is restricted to lowland areas where it is associated with three mosquito vectors: *Anopheles koliensis*, *An. farauti*, and *An. punctulatus*. The area has unstable malaria transmission. A household survey conducted in the same area in 2013 showed that malaria prevalence was 13.1%, with *P. falciparum* and *P. vivax* accounting for 5.9% and 6.0% of malaria, respectively.^11^

DHP is available, free of charge, from government health facilities and selected private-sector health facilities. Local protocol dictates that DHP is prescribed to patients with malaria confirmed by either rapid diagnostic test or microscopy. Local surveillance data showed that over 10 years, more than 485,000 doses of DHP have been administered to a population of 220,000 people. A cross-sectional survey undertaken in 2013 estimated that approximately 67% of individuals attend public health clinics, where DHP is available, for treatment of febrile illness.^11,12^ CQ and SP were available in a few private pharmacies and their use has declined to less than 5% following policy change in March 2006.

### Study design.

This was a prospective open-label drug efficacy study of dihydroartemisinin–piperaquine for *P. falciparum* and *P. vivax* malaria in children and adults with uncomplicated symptomatic malaria. The study was based on the 2009 World Health Organization antimalarial drug therapeutic efficacy tests^13^ with 42 days of follow-up.

### Patients.

Patients were eligible to enroll in the study if they were aged between 1 and 65 years, weighed more than 5 kgs, had a fever or history of fever in the preceding 24 hours, with slide-confirmed malaria with parasitemia of more than 1,000/μL asexual parasites for *P. falciparum* and more than 250/μL asexual parasites for *P. vivax*. Pregnant and lactating women, patients with signs of severe malaria,^14^ or those presenting with severe malnutrition or significant comorbidities were all excluded. Informed consent was obtained from patients aged more than 18 years old and in those less than 13 years old, from their parent or guardian. Children aged between 13 and < 18 years were asked for their assent in the presence of their parent or guardian. If patients were illiterate, consent was obtained in the presence of a literate witness. All patients were provided with an information sheet in Indonesian language.

### Study procedures.

On admission, a standardized questionnaire was completed to record demographic information and details of the patients’ symptoms, prior antimalarial medication, and their clinical examination. Axillary temperature was measured using a digital electronic thermometer. Venous or capillary blood was taken at enrolment for malaria smear, hemoglobin concentration, and parasite genotyping. Hemoglobin was measured using a portable photometer (HemoCue™ Hb201+; Ångelholm, Sweden). Glucose 6 phosphate dehydrogenase (G6PDd) status was measured at enrolment using the Fluorescent Spot Test method.^15^

Patients were examined daily for the first 3 days of DHP treatment and then weekly for 6 weeks. Patients were also encouraged to return to the clinic on any other day if they felt unwell. At each visit, a physical examination was performed, the symptom questionnaire completed, and blood taken for blood film examination and measurement of hemoglobin concentration.

Parasitemia was determined by microscopic examination of Giemsa-stained thick blood films, with parasites counted per 200 white blood cells (WBC) and peripheral parasitemia calculated assuming a white cell count of 7,300/μL. Slides were considered negative after review of 400 high-power fields. A thin smear was also examined to confirm parasite species and used for quantification if the parasitemia was greater than 200 per 200 WBC. All slides were read by a certified microscopist and cross-checked by a second experienced microscopist. In cases where readings were discordant, the slides were reread by a third microscopist and a consensus reached.

DNA from venous or capillary blood samples collected in ethylene diamine tetraacetic acid-coated Vacutainers or Microtainers^™^ at enrolment and again on the day of failure was extracted using the QIAamp DNA mini kit (Qiagen, Chadstone Centre, Victoria, Australia) as per the manufacturer’s instructions. Speciation was done by using an 18sRNA gene-based polymerase chain reaction (PCR) method for the detection of four different malaria species.^16,17^ For patients with recurrent *P. falciparum* infection, pre- and posttreatment isolates were genotyped using *msp1*, *msp2*, and *glurp* as described previously.^18,19^ The presence of polymorphisms in the propeller domains of the *P. falciparum* kelch (K13) gene, associated with artemisinin derivatives resistance, was determined by Sanger sequencing using the WWARN protocol.^20^ *Plasmepsin* 2–3 copy number variants, associated with piperaquine resistance, were also examined in all samples as described previously.^21–23^

### Treatment.

Dihydroartemisinin–piperaquine (containing 40 mg dihydroartemisinin and 320 mg piperaquine) was administered once daily for 3 days as a dose-per-weight regimen with a target dose of 2.25 and 18 mg/kg of dihydroartemisinin and piperaquine, respectively, according to the National Guidelines.^24^ In addition, patients with *P. vivax* malaria were prescribed primaquine (0.5 mg/kg/day for 14 days) commencing on day 28 of the study follow-up. All doses of DHP, but not primaquine, were administered under direct supervision and the patients observed for 30 minutes for adverse reactions or vomiting. Any patient vomiting their medication within this period was re-administered the same dose of DHP and observed for an additional 30 minutes. If the patient vomited again, then they were withdrawn from the study and hospitalized for artesunate intravenous therapy.

Patients with therapeutic failure within 28 days of initial treatment^25^ were treated according to local policy with a 7-day course of oral quinine (10 mg/kg body weight per dose, three times daily) plus doxycycline (2 mg/kg body weight per day, divided in two dose) in patients older than 8 years or clindamycin (5 mg/kg body weight per dose, three times per day) in children younger than 8 years. Patients with treatment failure after 28 days were retreated with DHP.

### Endpoints.

The primary endpoints of the study were the PCR-adjusted risk of recurrence of *P. falciparum* and the unadjusted risk of *P. vivax* recurrence. Secondary endpoints included the proportion of patients still parasitaemic on days 1, 2, and 3, posttreatment gametocyte carriage, and hematological recovery.

### Statistical analysis.

Data were double entered and validated using EpiData 3.02 software (EpiData Association, Odense, Denmark) and analysis performed using SPSS for Windows (SPSS Inc., Chicago, IL). The Mann–Whitney *U* test or Kruskal–Wallis method were used for nonparametric comparisons and Student’s *t* test or one-way analysis of variance for parametric comparisons. For categorical variables, percentages and corresponding 95% confidence intervals (95% CI) were calculated using Wilson’s method. Proportions were examined using χ^2^ with Yates’ correction or by Fisher’s exact test. The cumulative risk of failure was assessed by survival analysis using the Kaplan–Meier method. Anyone lost to follow up or representing with a different outcome were censored on their last day of follow-up and regarded as not being treatment failures. For determining the risk of *P. falciparum* recrudescence, all early treatment failures and homologous recrudescences were considered failures.

### Ethics.

The study was approved by the Eijkman Institute Research Ethics Commission, Jakarta, Indonesia (Ref: 014/EI-IRB/XII/2014), and the Human Research Ethics Committee of the NT Department of Health & Families and Menzies School of Health Research, Darwin, Australia (HREC-2015-2342). Informed consent was obtained from all adult participants and from parents of children. The trial was registered with the clinical trials website (http://www.clinicaltrials.gov/ct) as NCT 02353494.

## RESULTS

**Figure 1.**
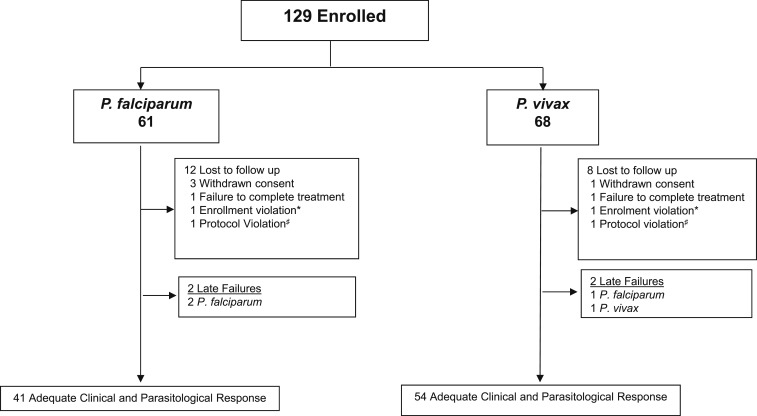
Trial Profile.

Between March 2015 and April 2016, 4,269 patients with fever were assessed by the health facility staff, of whom 1,039 (24%) were positive for malaria (513 *P. falciparum*, 479 *P. vivax*, and 27 mixed species malaria). Of these patients, 329 (32%) were screened by the study team for enrolment criteria. A total of 281 patients were eligible for the study, of whom 129 (45.9%) gave consent to participate in the study (61 *P. falciparum* and 68 *P. vivax* malaria) (see Figure 1 and Table 1). Of 48 patients excluded from the study, 46 were just visiting and two had comorbidities. Most of the patients who declined to be enrolled in the study were uncomfortable with making decisions without prior consultation with other family members. Molecular analysis at enrolment was possible in all but one patient, with the species of infection determined by PCR in concordance with that from microscopy.

**Table 1:** Baseline characteristics of uncomplicated malaria study patients

	*Plasmodium falciparum*	*Plasmodium vivax*	*P* value
Number of subjects enrolled	61	68	
Males % (*N*)	62.3 (38)	50 (34)	0.214
Age (years)			
Median [range], years	23.5 [4.3–63.9]	18.3 (1.8–50)	0.075
1 to < 5 years % (*n*)	1.6 (1)	1.5 (1)	
5 to < 15 years % (*n*)	18.0 (11)	30.9 (21)	0.241
15 to 65 years % (*n*)	80.3 (49)	67.6 (46)	
Temperature (°C)			
> 37.5% (*n*)	52.5 (32)	48. (33)	0.725
Mean (95% CI)	37.8 (37.5–38.1)	37.8 (37.5–38.2)	0.033
Hemoglobin (g/dL)			
Mean (95% CI)	13.8 (13.1–14.5)	13.2 (12.6–13.8)	0.699
< 10 g/dL % (*n*/*N*)	5.2 (3/58)	6.2 (4/65)	1.000
Geometric mean (95% CI) parasite count per µL blood	12,088 (9,897–14,764)	9,897 (7,331–10,938)	0.994

CI = confidence intervals.

Five patients (3.9%) vomited their medication after the first dose, but tolerated the repeat dose. The median total dose of dihydroartemisinin administered was 7 mg/kg (range 5.4–10.6) and that of piperaquine was 57 mg/kg (range 43.2–84.7). In total, one male patient with *P. falciparum* was G6PD deficient and another male with *P. vivax* had intermediate G6PD activity; primaquine was not prescribed to either of these patients.

### Therapeutic response.

In total, four patients had recurrent parasitemia during the follow-up period (three with *P. falciparum* and one with *P. vivax*). The overall unadjusted efficacy at day 42 was 98.2% [95% CI: 90.3–100].

Of the 61 patients with *P. falciparum* infection, 43 (70.5%) completed 42 days of follow-up and 41 had an adequate clinical and parasitological response. Two patients had asymptomatic recurrent *P. falciparum* parasitemia recorded on day 42: a 15-year-old female treated with 48.8 mg/kg of piperaquine with reinfection and a 52-year-old male treated with 53.3 mg/kg of piperaquine with recrudescent infection. The unadjusted efficacy for *P. falciparum* at day 42 was 95.3% [95% CI: 84.2–99.4] and after adjusting by PCR, this rose to 97.7% [95% CI: 87.4–99.9]. Of the 68 patients with *P. vivax* infection, 56 (82.3%) completed follow-up. Two patients had recurrent parasitemia on day 35: a 2-year-old boy with *P. vivax* infection in whom the parasite genotype could not be determined because insufficient blood was taken at enrolment and an 18-year-old female with symptomatic *P. falciparum* infection. The unadjusted efficacy for *P. vivax* at day 42 was 98.2% [95% CI: 90.4–100]; Table 2.

**Table 2:** The cumulative risk of treatment failure according to initial species of infection

	*Plasmodium falciparum* [95% CI]	*Plasmodium vivax* [95% CI]
Early treatment failure %	0	0
Risk of treatment failure at day 28	0	0
Risk of recurrence with the same species at day 42	4.7 [0.6–15.8]	1.8 [0–9.7]
Risk of recurrence by day 42—PCR adjusted	2.3 [0.1–12.3]	–*

CI = confidence intervals; PCR = polymerase chain reaction.

* One recurrent infection with *P. vivax*, which had insufficient blood to genotype.

Parasite clearance was rapid following treatment in both *P. falciparum* and *P. vivax* malaria. Within 24 hours, 62.7% (37/59) of patients with *P. falciparum* and 74.6% (50/67) of those with *P. vivax* were aparasitemic, and by 48 hours, this had risen to 98.2% (56/57) and 96.9% (63/65), respectively. None of these patients were parasitemic on day 3.

### Gametocyte carriage following treatment.

Gametocytemia was present at enrolment in 3.3% (2/61) of patients infected with *P. falciparum* and 26.5% (18/68) with *P. vivax* malaria. The proportion of patients with patent gametocytemia in those with *P. falciparum* infection was 10.2% (6/59) at 24 hours, 12.3% (7/57) at 48 hours, and 5.9% (3/51) at day 7. In patients with *P. vivax* malaria, the proportion with gametocytes fell to 6% (4/67) by 24 hours, 4.6% (3/65) at 48 hours, and 1.5% (1/65) at day 7. One patient had *P. falciparum* gametocytes detected at day 14, and one patient had *P. vivax* gametocytes detected at day 21. None of the patients had gametocytes after day 21.

### Hemoglobin reduction after treatment.

Mean hemoglobin concentration at enrolment was 13.8 g/dL [95% CI: 13.1–14.5] in patients with *P. falciparum* and 13.2 g/dL [95% CI: 12.6–13.8] in those with *P. vivax* (Table 1). The mean reduction in hemoglobin concentration at day 14 was 1.65 g/dL [95% CI: 1.14–2.15] in *P. falciparum* and 0.83 g/dL [95% CI: 0.31–1.33] in *P. vivax* malaria (*P* = 0.661).

### Adverse events.

No severe adverse events were reported associated with treatment. Twenty-four hours following treatment, 21.8% (27/124) of the patients had nausea (8), dyspepsia (2), dizziness (13), urticaria (1), and weakness (3). At 48 hours, 9.9% (12/121) patients had mild nausea. All symptoms were resolved quickly. One case with urticaria was not associated with DHP, but due to mild food allergy.

### K13 genes and *plasmepsin* 2–3 amplifications in *P. falciparum*.

Molecular analysis could be undertaken in all *P. falciparum* samples, with no mutations in the K13 gene (0/65) or increased copy number of *plasmepsin* 2–3 (0/74).

## DISCUSSION

In March 2006, Indonesia was one of the first countries to adopt a unified first-line policy of ACT for the treatment of uncomplicated malaria due to any species of malaria. It was also the first country to adopt DHP as its preferred choice of ACT, a decision made in view of its high efficacy and prolonged posttreatment prophylaxis associated with piperaquine, the slowly eliminated partner drug.^4^ Over the ensuing decade, DHP has been used extensively throughout the country. This recent therapeutic efficacy study in the eastern province of Papua reassuringly confirms that, despite the high prevalence of artemisinin and emerging piperaquine resistance in the GMS, DHP retains high efficacy against uncomplicated *P. falciparum* and *P. vivax* malaria after 9 years of use in this region.

The PCR-adjusted efficacy in patients with *P. falciparum* malaria was greater than 97%, similar to that reported in 2005.^4^ The efficacy of DHP against *P. vivax* was almost 100%, with only one of 56 patients (1.8%) having a recurrent infection on day 35, compared with an efficacy of 90% reported in 2005.^4^ Compared with our original efficacy study in 2005, the baseline parasitemias in the present study were 3–4 fold higher and the gametocyte carriage 2–7 fold lower. The high gametocyte carriage at enrolment in 2005 likely reflects the poor efficacy of the first-line treatment before policy change in 2006, with almost 20% of the patients reporting a history of malaria in the preceding month.^3^ With the wide deployment of highly efficacious treatment, the proportion of patients partially treated and reporting recent malaria has fallen substantially.^11,12^

Patients were only assessed once per day precluding precise estimation of the slope of the parasite clearance curves.^26^ However, the proportion of parasitemic patients fell rapidly, with more than 98% aparasitemic by 48 hours and none of the patients remaining parasitemic at 72 hours. Molecular analysis confirmed that none of the parasites had K13 gene mutations that have been associated with artemsinin resistance or amplification of the *plasmepsin* 2–3 gene cluster that have been associated with piperaquine resistance.

Resistance to artemisinin derivatives for the treatment of *P. falciparum* malaria was first described in western Cambodia in 2008^27^ and has been reported subsequently in Thailand, Myanmar, Laos, and Vietnam.^6,28,29^ In Cambodia, where the prevalence of artemisinin resistance is greatest, recent clinical trials have revealed that the efficacy of DHP for *P. falciparum* has fallen to less than 80%, with almost half of the patients still parasitemic at 72 hours.^30^ Reassuringly, in the same study DHP retained good efficacy against *P. vivax*.^30^

Piperaquine was first synthesized in the 1960s and used extensively in China and Indochina as prophylaxis and treatment over the next 20 years. Within a decade, high rates of treatment failure were observed and piperaquine monotherapy was discontinued. The drug was removed from the pharmacopeia until it was reintroduced in 1990s in combination with dihydroartemisinin, trimethoprim, and primaquine phosphate.^31^ The recommended combination has subsequently rationalized to DHA plus piperaquine which has been available outside of China since the start of the millennium. The significant mismatch in the pharmacokinetic profiles of these two drug components has raised concerns of exposing parasites to subtherapeutic concentrations of piperaquine monotherapy in the 2–6 weeks following treatment, particularly where the risk of reinfection is high. It is feared that this mismatch will foster the emergence and spread of drug-resistant parasites. Indeed in this respect, it is interesting to note that in an area where artemisinin resistance has emerged the efficacy of the combination fell quickly.^8,21,30^

The results from this Papuan study are reassuring and suggest that in the absence of artemisinin resistance, the ACT regimen protects against de novo emergence of resistance. In Cambodia, amplification of the *plasmepsin* 2–3 gene cluster has been identified as an important molecular determinant of piperaquine resistance in *P. falciparum*.^22^ Our molecular analysis reveals that neither of these polymorphisms is present in Timika.

It is likely that the development of artemisinin resistance in Cambodia has been driven by high rates of self-treatment via private sector and unregulated use of antimalarial agents, including poor ACT quality. In Indonesia, DHP procurement is highly regulated by the Indonesian Ministry of Health, and the drug is only available at government health facilities and selected private-sector facilities, which are able to confirm the diagnosis of malaria by microscopy or rapid diagnostic test. Hence, DHP is generally only prescribed to those with confirmed malaria and this degree of control may have contributed to the sustained efficacy of the drug in this region.

In conclusion, despite intense use of DHP over almost a decade in a region of mesoendemic malaria, there is no evidence for the presence of either artemisinin or piperaquine resistance and the combination regimen retains excellent efficacy. However, in view of the highly mobile population and potential spread of artemisinin resistance from the GMS, vigilance for declining efficacy is warranted.
